# Integrated Lipidomic and Metabolomics Analysis Revealing the Effects of Frozen Storage Duration on Pork Lipids

**DOI:** 10.3390/metabo12100977

**Published:** 2022-10-16

**Authors:** Xiaohui Feng, Jing Li, Longchao Zhang, Zhenghua Rao, Shengnan Feng, Yujiao Wang, Hai Liu, Qingshi Meng

**Affiliations:** 1 A State Key Laboratory of Animal Nutrition, Institute of Animal Sciences, Chinese Academy of Agricultural Sciences, Beijing 100193, China; 2 School of Agricultural Sciences, Zhengzhou University, Zhengzhou 450001, China; 3 College of Food Science and Technology, Hunan Agricultural University, Changsha 410128, China; 4 Beijing Heiliu Animal Husbandry Technology Co., Ltd., Beijing 102211, China

**Keywords:** lipidome, metabolome, lipid oxidation, pork, freezing process

## Abstract

Frozen storage is an important strategy to maintain meat quality for long-term storage and transportation. Lipid oxidation is one of the predominant causes of the deterioration of meat quality during frozen storage. Untargeted lipidomic and targeted metabolomics were employed to comprehensively evaluate the effect of frozen duration on pork lipid profiles and lipid oxidative products including free fatty acids and fatty aldehydes. A total of 688 lipids, 40 fatty acids and 14 aldehydes were successfully screened in a pork sample. We found that ether-linked glycerophospholipids, the predominant type of lipids, gradually decreased during frozen storage. Of these ether-linked glycerophospholipids, ether-linked phosphatidylethanolamine and phosphatidylcholine containing more than one unsaturated bond were greatly influenced by frozen storage, resulting in an increase in free polyunsaturated fatty acids and fatty aldehydes. Among these lipid oxidative products, decanal, cis-11,14-eicosenoic acid and cis-5,8,11,14,17-dicosapentaenoic acid can be considered as potential indicators to calculate the freezing time of unknown frozen pork samples. Moreover, over the three-month frozen storage, the first month was a rapid oxidation stage while the other two months were a slow oxidation stage.

## 1. Introduction

Pork is one of the most globally consumed meats; the average global consumption was about 112.3 million metric tons per year from 2018 to 2020 (OECD/FAO, 2021). Especially in China, pork consumption has increased from 23.2 kg per person in 2020 to 33.9 kg in 2021 (USDA). To meet the increasing demands and provide stable supply, pork import and strategic storage are very necessary for big consumer countries. How to safely deliver high-quality pork to frequently distant end-users and long-term storage are significant concerns for the pork industry [1]. Frozen storage is one of the widely utilized methods to preserve meat quality for distribution and long-time storage. Its advantages include slowing down microbial reproduction and suppressing autolytic reaction, thereby effectively prolonging the shelf life of meat [2,3]. However, the meat still undergoes the oxidation spoilage process, resulting in the deterioration of meat quality.

Frozen storage can influence the properties of meat including pH, shear force, electrical conductivity, and meat color [4]. Many studies have examined the alteration of these physicochemical properties of meat subjected to frozen storage. Medic et al. reported that during frozen storage, a significant increase in the pH of pork in lion, ham and belly rib was observed compared with that of unfrozen ones [5]. The increase in pH can be explained by protein denaturation, which involves proteolysis followed by the release of free amino acids and the accumulation of amine [6]. A similar trend was observed for pork *M. Longissimus thoracis et lumborum* [7]. As frozen storage time was prolonged, the shear force of the pork declined [5]. This phenomena was observed for beef [2,8] and lamb [9]. The structure damage is attributed to ice crystal formation during refrigerator storage, in which intercellular ice crystals separate the muscle fiber and intracellular ice crystals lead to muscle cell rupture. Furthermore, this change could be caused by postmortem proteolysis. Electrical conductivity, which was considered as an indicator of meat freshness, increased remarkably along with frozen time extension, suggesting that it was negatively correlated with meat freshness. This can be due to the rise in the content of conductivity substances as meat tissue degrades [10,11]. The color of the meat, which was characterized using the lightness (L^*^), redness (a^*^), and yellowness (b^*^), is another meat freshness indicator. Lee et al. indicated that the total color difference (ΔE) of pork loin increased with frozen treatment regardless of time because of higher L^*^ and b^*^ [12]. Li et al. also showed that the a^*^ value of the pork patties decreased while the L^*^ and b^*^ values increased, leading to higher light reflection [13]. The increase in L^*^ value was demonstrated for both boiler breast fillets [14] and lamb [15] subjected to the freezing process, which can be attributed to an increase in the amount of free water release [8]. The decrease in meat a^*^ can be attributed to the loss of myoglobin [16] whereas the increase in meat b^*^ can be due to the high susceptibility of myoglobin molecules to oxidization to metmyoglobins, showing a dark color. The frozen temperature decreased the reduction activity in enzymes such as NADPH, leading to the increase of myoglobin oxidation to form oxymyoglobin, showing a bloomed color. Lipid pro-oxidation was another factor that initiated the myoglobin oxidation [17,18,19]. Herein, a similar trend in the alterations of physiochemical properties was observed for frozen meat, including an increase in pH, electrical conductivity and total color difference but a decrease in shear force.

Lipid oxidation has been acknowledged as one of the major causes of meat quality deterioration [20]. Muscle tissue is highly susceptive to oxidization because it contains higher levels of phospholipids including a higher amount of polyunsaturated fatty acids (PUFAs) [2,21]. The oxidation of lipids is initiated by free radicals or enzymes through a radical chain reaction to produce alkyl radicals, which are chemically unstable. These alkyl radicals are further oxidized to form lipid-hydroxide (•OOH) intermediates, which undergo propagation and terminate reactions to generate hydroperoxides and aldehydes [22,23,24]. The degree of lipid oxidation is influenced by endogenous factors (heme, nonheme iron and tissue enzyme) and exogenous factors (storage temperature and storage time) [25]. Conventionally, the extent of oxidative damage is evaluated using primary (e.g., hydroperoxide) and secondary oxidative products (e.g., thiobarbituric acid reactive species (TBARs) and aldehydes). For example, Coombs et al. [26] studied the influence of frozen time on esterified fatty acid (FAs) profiles and lipid oxidation in lamb and the results showed that TBARs increased over frozen storage time, suggesting a rise in lipid oxidation. Similar phenomena were also observed for pork [5], peeled pacific white shrimp [18,27], lamb and beef during frozen storage [5,26,27]. These conventional strategies can provide some information on the progress of lipid oxidation; however, they could not provide details on reactive intermediates or the corresponding lipid oxidative products. Further, the detailed correlation between lipid oxidation and lipid-derived products in muscle tissues still requires further investigation.

Lipidomic analysis provides a comprehensive way to reveal the profile of lipids, their compositions, and dynamic change and functions in tissues, as well as how specific lipid species differ in the linkage between fatty acid and backbone, and the number and location of unsaturated bonds [28]. Mass spectrometry (MS)-based lipidomics has been widely utilized to study the variations in lipid metabolism and their compositions to distinguish lipid species and illuminate the functional roles of specific lipids [29]. Untargeted metabolomics can provide a comprehensive profile of all measurable analytes including chemical unknowns based on the existing database; however, accuracy is low. Targeted metabolomics focus on measuring groups of analytes of interest as well as specific metabolite groups [30]. Compared with nontargeted metabolism, targeted metabolism provides enhanced detection sensitivity, high data accuracy and reliability that are achieved through standard products [31].

In the current study, we investigated the effect of frozen storage on the extent of lipid oxidation through the variations in lipid species, FAs structure and compositions in lipid fractions using non-targeted lipidomics. Targeted metabolism was utilized to reveal the changes in fatty acids and fatty aldehydes produced from lipid oxidation. The correlation study between lipid species, lipid compositions and the corresponding products revealed the detailed relationship between lipidomic profiles and lipid oxidation products induced by frozen stress occurring in pork meat.

## 2. Material and Methods

### 2.1. Chemicals

The fatty acid (FA) standards were all of analytical grade and from ANPEL Laboratory Technology Inc. (Shanghai, China). Fatty aldehyde standards were supplied by Aladdin Industrial Co., Ltd (Shanghai, China). Isopropanol, acetonitrile, methanol, N, N-dimethylformamide, dichloromethane, as well as ethanol, were all high-performance liquid chromatograph (HPLC) grade and purchased from Thermal Fisher Scientific (Shanghai, China). N-(3-Dimethylamnopropyl)-N’-ethylcarbodiimide hydrochloride (EDC), 1-hydroxyl-7-azabenzotriazole (HOAt), ammonium acetate ((NH_4_)_2_Ac), hydrochloride acid (HCl, 36.5~38 wt%), and sodium hydroxide (NaOH) were all of analytical grade and obtained from Energy-Chemical (Beijing, China). 1,5-dipentadecanoyl-2-(9Z-octadecenoyl)-glycerol(d5) (TG 15:0/18:1/15:0 (d5)), Dodecanoic acid(d23), and Ceramide (d18:1/2:0) were supplied by Avanti Polar Lipids (Birmingham, AL, USA). Both 5-(di(methyl-^12^C)amino-^14^N) isoquinoline-1-carbohydrazide (DMAQ-^12^C/^14^N) and 5-(di(methyl-^13^C)amino-^15^N) isoquinoline-carbohydrazide (DMAQ-^13^C/^15^N) derivatization reagents were prepared in-house [32].

### 2.2. Preparation of Fatty Acids and Aldehydes Internal Standard Solution (IS)

A standard solution containing 40 FAs was made in ethanol at a concentration of 2 mg/L. The obtained standard solution was derivatized by DMAQ-^13^C/^15^N using the reported method [33,34]. The mixed working solution containing 14 fatty aldehydes were prepared in methanol at a concentration of 10 mg/L, which was derivatized by DMAQ-^13^C/^15^N [32].

### 2.3. Sampling

All animal procedures undertaken in this study were approved by the Animal Care and Use Committee of the Institute of Animal Sciences of the Chinese Academy of Agricultural Sciences (Beijing, China) with permission number, IAS2022-38. Following an 18-h overnight fast with free access to water, five pigs were randomly selected, then electrically stunned, bled, dehaired, peeled, eviscerated and spilt down following the requirements of the China Council on Animal Care at Beijing Heiliu Stockbreeding Technology Co., Ltd. (Beijing, China). A total of five pig longissimus thoracis (LT) samples, each weighing around 500 g, were obtained from pig carcasses within 24 h after slaughter. The samples were stored in a cool room at 4 °C for 12 h. After chilling, each sample was equally sectioned into seven proportions. One of the seven proportions, named T1, was immediately used to extract lipids, fatty aldehydes, and free and esterified FAs. The rest of the blocks of meat were stored at −20 °C and the frozen samples were collected after 10 days (T2), 20 days (T3), 30 days (T4), 45 days (T5), 60 days (T6) and 90 days (T7) of storage. At the end of each freezing stage, the collected meat sample was thawed for 12 h in a refrigerator at 4 °C until the core temperature reached 0–2 °C, which was determined in real time using a thermometer. The adipose and connective tissue was removed, followed by blending for 5 min with a KitchenAid mixer.

### 2.4. Untargeted Lipidomic Analysis

Lipid extraction was carried out following the standard method reported by Folch, Lees, and Stanley [35,36]. In brief, pork samples were homogenized in ice-cold MeOH containing 1 ug/mL of TG15:0/18:1/15:0 (d5). Then dichloromethane and deionized water were added in sequence with the final volumetric ratio of dichloromethane: methanol: deionized water to 4:2:1. The organic phase was collected using centrifugation. The extraction and centrifugation steps were repeated twice. All the liquid phase samples were dried under nitrogen. The dry pellets were stored at –80 °C and redissolved in dichloromethane to methanol with a volumetric ratio of 2 to 1. The quality control sample (QC) was made by polling an aliquot of all meat samples.

Lipid profiling was conducted using a modular 1290 infinity high-performance liquid chromatograph (Agilent Technologies, Waldbronn, Germany) connected to a quadrupole time-of-flight mass spectrometer (AB Sciex 6600, Framingham, MA, USA). The lipids were chromatographically separated on reverse-phase chromatography equipped with an ACQUITY C8 BEH column (2.1 × 100 mm, 1.7 μm particle size, Waters, Milford, MA, USA) in both positive and negative ion mode. Mobile phase A was the mixture of methanol, acetonitrile, and deionized water (1:1:1, *υ*:*υ*:*υ*) while mobile phase B consisted of isopropanol. Both of them contained 5 mM (NH_4_)_2_Ac. The gradient elution procedure was programmed as follows: 0–2 min, 15–40% B; 2–6.5 min, 40–50% B; 6.5–7 min, 50–58% B; 7–13 min, 58–62% B; 13–14 min, 62–70% B; 14–14.5 min, 70% B; 14.5–14.6 min, 70–15% B; 14.6–16.0 min, 15% B. The total runtime was 16 min with a flow rate of 300 μL/min. The column temperature was set at 50 °C, and an injection of 3 μL was applied. Lipids were introduced by an autosampler with the temperature set to 4 °C. The MS parameters for lipid extracts were as follows: the ion-spray (IS) voltage of 5.0 kV in positive mode and 4.5 kV in negative mode; the turbo source gun temperature of 500 °C; and curtain gas (CUR) of 35 psi.

In this study, the comprehensive lipid profile of pork samples was carried out by the nontargeted lipidomic method on the UPLC-TripleTOF 6600 platform with in-house developed lipidomic workflow. A vast amount of information quantitatively described the longitudinal and sequential alterations in the compositions of different lipid species during the freezing process. All the peaks in raw data were detected, aligned, and identified using MS-DIAL4.80 coupled with LipidBlast (version 68, 21 April 2022) [37,38]. A total of 1,042 and 987 lipids were identified in positive and negative mode, respectively (Supplementary Tables S1 and S2). Of these identified lipids, the lipids with only MS1 matched and the signal-to-noise ratio (S/N) lower than 10 was filtered out. For lipids that had multi-adduct peaks or those detected in both positive and negative mode, the peaks with the highest S/N were chosen for subsequential quantitative analysis. To reduce the run-to-run variability, the MS intensities were normalized by the LOWESS using QC samples [39].

### 2.5. Fatty Acids Analysis

The FAs extraction and analysis were carried out according to our reported method [33,34]. For free FA extraction, the meat sample was homogenized in ice-cold acetonitrile containing a proper concentration of DMAQ-^13^C/^15^N-FA IS. The suspension was centrifuged, and the supernatant collected. For total FA extraction, the meat sample was homogenized in 80% methanol containing 0.5 M NaOH and an appropriate concentration of DMAQ-^13^C/^15^N-FA IS. The mixture was heated to 80 °C. After 3 h, the suspension was cooled to room temperature, adjusted to neutral condition using 2 M HCl and diluted to 250 mL using methanol.

The obtained FA extract was derivatized using DMAQ-^12^C/^14^N. Briefly, an aliquot of the FA extract was mixed with the derivatization solution containing 750 mM EDC, 20 mM HOAt and 20 mM DMAQ-^12^C/^14^N. The derivatization reaction was undertaken at 20 °C for 30 min, followed by the addition of 10% formic acid. The mixture was diluted in acetonitrile. FA analysis was carried out on ACQUITY Ultra-Performance Liquid Chromatography (Waters, Milford, MA, USA) coupled with triple-quadrupole mass spectrometry (QTRAP 6500, SCIEX, Framingham, MA, USA). Chromatographic separation was achieved using an Eclipse Plus C8 RRHD column (2.1 × 100 mm, particle size: 1.8 μm, pore size 95 Å, Agilent Technologies, Waldbronn, Germany). Mobile phase A was deionized water and mobile phase B composed of isopropanol and acetonitrile (*υ*:*υ* 1:1). Both Mobile phase A and B contained 0.1% formic acid. The 20-min gradient conditions were: 0–1.5 min (30–60% B), 1.5–11.0 min (60–75% B), 11.0–15.0 min (75–100% B), 15.0–18.0 min (100% B), 18.0–18.1 min (100–30% B) and 18.1–20.0 min (30% B). The flow rate was set at 400 μL/min. The MS parameters were an IS voltage of 5.5 kV, turbo source gun temperature of 500 °C and CUR of 30 psi.

### 2.6. Fatty Aldehyde Analysis

Fatty aldehydes were extracted and derivatized according to the previous reported method [32]. In brief, the pork sample was homogenized in ice-cold methanol containing a proper concentration of DMAQ-^13^C/^15^N-aldehydes. The supernatant was collected by centrifugation. Twenty microliters of meat extract and 20 μL of 5 mM DMAQ-^12^C/^14^N solution was added into 60 μL of the mixture of methanol and water (1:1, *υ*:*υ*) containing 0.1% formic acid. The reaction mixture was incubated at 20 °C for 15 min, and then concentrated under vacuo and reconstructed in 1 mL of acetonitrile. Aldehydes analyses were performed using an ACQUITY^TM^ UPLC equipped with a 6500 QTRAP Triple Quadrupole Mass Spectrometer (SCIEX, Framingham, MA, USA). Chromatograph separation was achieved via ACQUITYTM Premier BEH C18 column (2.1 × 150 mm, 1.7 μm particle size and 130 Å pore size). Mobile phase A and B were deionized water and acetonitrile, respectively. Both contained 0.1% formic acid. Gradient elution (t = 0 min, 25% B; t = 2 min, 28% B; t = 5 min, 60% B; t = 8 min, 100% B: t = 11 min, 100% B; t = 11.1 min, 25% B; t = 12 min, 25% B) was carried out at 300 μL/min. The column temperature was set at 45 °C. The MS parameters were set as follows: IS voltage of 5.5 kV, turbo source gun temperature of 450 °C and CUR of 30 psi.

### 2.7. Statistical Analysis

All the lipid peaks in the raw data were detected, aligned and identified using MS-DIAL4.80. MultiQuant 3.0 software (SCIEX, Framingham, MA, USA) was applied for the calculation of FAs and fatty aldehyde concentrations. At each freezing stage, five biological replicates were performed, and the results were represented as mean ± standard derivation (std). Multivariate statistical analyses including PCA and OPLS-DA analysis were executed using Simca 14.1 (Umertric, Umea, Sweden). Statistical analysis was carried out using SPSS 23.0 software (SPSS, Inc. Chicago, IL, USA) using post HOC multiple comparison with one-way ANOVA. Correlation analysis was carried out using Pearson and statistical significances were considered if *p* was lower than 0.05. Figures were drawn by GraphPad Prism 9.0 and R 4.1.2 installed on ggplot2.

## 3. Results

### 3.1. Untargeted Lipidomic Profiling of the Changes in Pork during Freezing Storage

**Figure 1:**
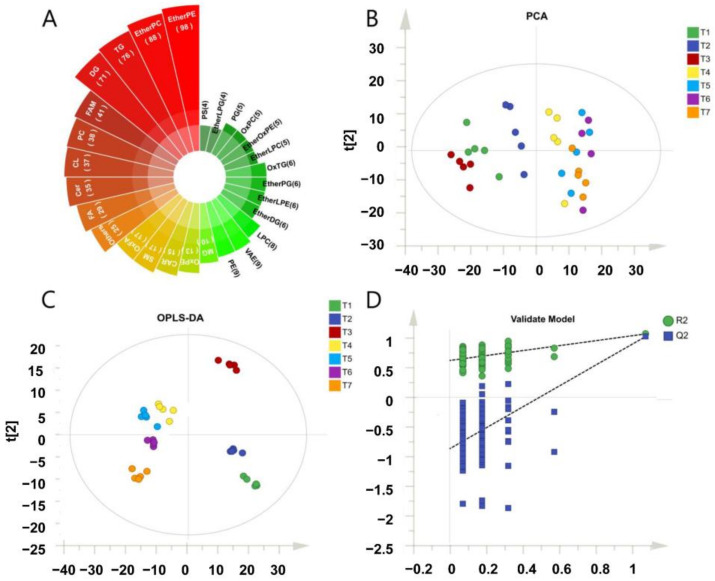
Lipidomic analysis revealed that the frozen storage duration had a significant influence on the lipid profiles of pork. (**A**) The 28 identified lipid species in pork. (**B**) The principal component analysis (PCA) and (**C**) the supervised orthogonal partial least squares discriminate analysis (OPLS-DA) score plot of the confirmed lipids in pork. (**D**) The validation mode of OPLS-DA. EtherPE, ether-linked phosphatidylethanolamine; EtherPC, ether-linked phosphatidylcholine; TG, triacylglycerols; DG, diacylglycerol; FAM, fatty amides; PC, phosphatidylcholines; CL, cardiolipin; Cer, ceramides; FA, free fatty acids; OxFA, oxidized fatty acid; SM, sphingomyelin; CAR, acylcarnitine; OxPE, oxidized phosphatidylethanolamine; MG, monoacylglycerol; PE, phosphatidylethanolamine; VAE, vitamin A fatty acid ester; LPC: lysophosphatidylcholine; EtherDG, ether-linked diacylglycerol; EtherLPEether-linked lysophosphatidylethanolamine; EtherPG, ether-linked phosphatidylglycerol; OxTG, oxidized triacylglycerols; EtherLPC, ether-linked lysophosphatidylcholine; EtherOxPE, ether-linked oxidized phosphatidylethanolamine; OxPC, oxidized phosphatidylcholine; PG, phosphatidylglycerol; EtherLPG, ether-linked lysophosphatidylglycerol; PS, phosphatidylserine.

The lipidomic profile of pork meat is affected by type, origin, processing method, storage temperature and time. In this study, a total of 688 lipids were employed to analyze the changes in lipids in pig LT (Supplementary Tables S3–S5), and they can be assigned to 28 subclasses according to the LIPID MAPS classification system [40]. The top 10 of the highest number of the lipid species in pig muscle were ether-linked phosphatidylethanolamine (EtherPE), ether-linked phosphatidylcholine (EtherPC), triacylglycerols (TG), diacylglycerol (DG), fatty amides (FAM), phosphatidylcholines (PC), cardiolipin (CL), ceramides (Cer), free fatty acid (FA), and oxidized fatty acid (OxFA) (Figure 1A). To reveal the lipid changes along with frozen storage, multivariant statistical analyses, including unsupervised principal component analysis (PCA) and supervised orthogonal partial least squares discriminate analysis (OPLS-DA) were utilized, respectively, and shown in Figure 1B,C. According to the PCA results, the samples from the first month (T1 to T4) were clustered away from that of the second month and third month (T5 to T7). Moreover, T4 to T7 samples were clustered together. The results indicated that the changes in lipids mainly happened in the first month of frozen storage. OPLS-DA score plots for the first two components showed that T4 to T7 samples could be clustered into different subgroups. The results suggested that there was a slight deviation as frozen storage was prolonged. The R2Y and Q2Y values, which indicated the fitness and predictability of the model, were 0.879 and 0.968, respectively. Next, the Y variable was randomly permuted to 100 times to rebuild the statistical model. We observed the trend of the predictive power and goodness of fit of the data in the model. The validation plot (Figure 1D) strongly indicated that the original model was valid. The Q2 regression line had a negative intercept, and all permuted R2 values to the left of the intercept were lower than the original point to the right. Thus, the OPLS-DA model was robust, and overfitting did not occur. The results also suggested that the LC-MS-based lipidomic approach was applicable for the comprehensive lipidomic analysis of pork meat.

**Figure 2:**
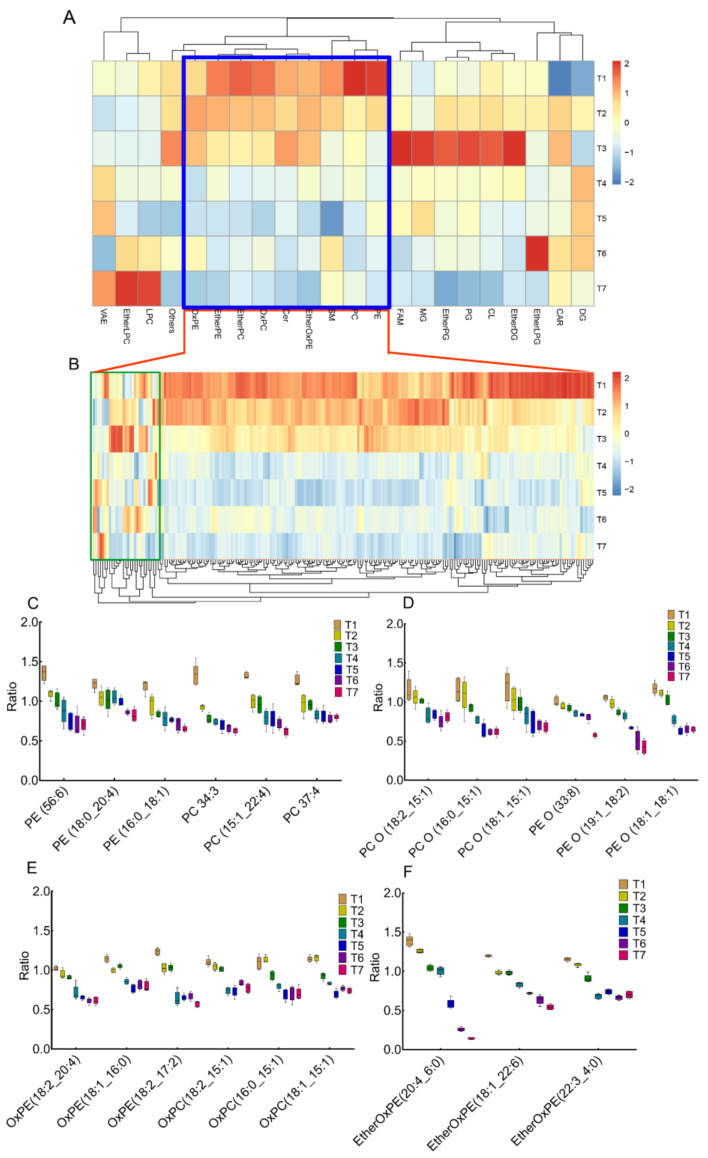
Lipid profiles in the LT of pork during frozen storage. (**A**) The change in different lipid subclasses along with a frozen period. In the blue box, the GPLs gradually decreased along with the freezing process; (**B**) The changing pattern of each lipid belongs to GPLs. Most of the GPLs gradually decreased with prolonged frozen storage, except for some PE lipids (highlighted with green box). (**C**–**F**) Representative lipid molecules with top-three-highest fold changes and *p*-value lower than 0.05. Data represented as mean ± std (*n* = 5). VAE, vitamin A fatty acid ester; EtherLPC, ether-linked lysophosphatidylcholine; LPC, lysophophatidylcholine; OxPE, oxidized phosphatidylethanolamine; EtherPE (PE O), ether-linked phosphatidylethanolamine; EtherPC (PC O), ether-linked phosphatidylcholine; OxPC, oxidized phosphatidylcholine; Cer, ceramides; EtherOxPE, ether-linked oxidized phosphatidylethanolamine; SM, sphingomyelin; PC, phosphatidylcholine; PE, phosphatidylethanolamine; FAM, fatty amides; MG, monoacylglycerol; EtherPG, ether-linked phosphatidylglycerol; PG, phosphatidylglycerol; CL, cardiolipin; EtherDG, ether-linked diacylglycerol; EtherLPG, ether-linked diacylglycerol; CAR, acylcarnitine; DG, diacylglycerol; GPL, glycerophospholipids.

To address the influence of frozen storage on the pig LT lipidomic, we firstly compared lipid subclasses using post HOC multiple comparison with one-way ANOVA. The results indicated that most lipids species were significantly changed (*p* < 0.05), except TG, PS, OxTG, OxFA, FA, EtherLPE, and CAR (Table 1). Then, the significant changed lipid species were utilized to reveal the changing tendency along with frozen storage. As shown in Figure 2A, the glycerophospholipids (GPLs), including PE, PC, EtherPE, EtherPC, EtherOxPE, OxPE and OxPC, gradually decreased along with the freezing process, which was highlighted using the blue box. A similar trend was indicated for Cer and SM. The effect of frozen duration on individual lipids was also analyzed based on all identified lipids; results shown in Figure 2B and Table S4. Most of the GPLs gradually decreased with frozen storage over time, except for some PE lipids highlighted in the green box. Of these significant GPLs, more than one unsaturated bond was demonstrated in the fatty acid structure of lipids. They could be grouped in ether-linked GPL (170), ester-linked GPL (31) or oxidized GPL (20). The lipid molecules with the top three highest fold change and *p*-value lower than 0.05 were summarized in Figure 2C–F. Thus, the lipid oxidation on ether-linked GPL contributed to the variations in lipids during frozen storage.

### 3.2. The Effect of Frozen Storage on Total and Free FAs in Pork

**Figure 3:**
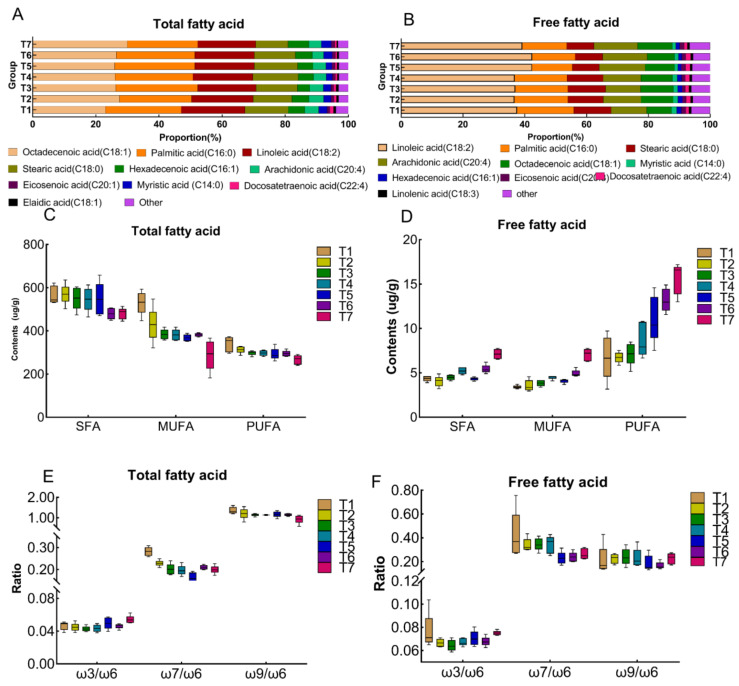
The effect of frozen storage on the profiles of total FAs and free FAs. The proportion of top 10 total FAs (**A**) and free FAs (**B**) in pork during frozen storage; The changes of SFA, MUFA and PUFA contents in (**C**) total FAs and (**D**) free FAs along with frozen storage; The oxidation rate for ω3/ω6, ω7/ω6 to ω9/ω6 in (**E**) esterified FAs and (**F**) free FAs subjected to frozen storage. SFA, saturated fatty acid; MUFA, monounsaturated fatty acids; PUFA, polyunsaturated fatty acids. Data represented as mean ± std (*n* = 5).

To reveal the changing trend of FAs produced from these GPLs, targeted metabolomics was utilized and discussed. We quantified total FAs after saponification (Table S6) and free FAs without saponification (Table 2) in pork subjected to frozen storage. In Figure 3A, the top 10 of total FAs were cis-9-octadecenoic acid, palmitic acid, linoleic acid, stearic acid, cis-9-hexadecenoic acid, arachidonic acid, myristic acid, cis-8,11,14-eicosenoic acid, cis-11-eicosenoic acid, cis-7,10,13,16-docosatetraenoic acid and elaidic acid. These FAs accounted for about 97% of total FAs in all pork samples, which well agreed with previous reports [41,42]. The content of cis-9-octadecenoic acid, palmitic acid and linoleic acid in aged meat decreased by 46.7%, 15.1%, and 23.7%, respectively, after three-month frozen storage. Compared with total FAs, the major compositions of free FAs were different. As shown in Figure 3B, the top 10 of free FAs were linoleic acid, palmitic acid, stearic acid, arachidonic acid, cis-9-octadecenoic acid, myristic acid, cis-9-hexadecenoic acid, cis-11,14,17-eicosenoic acid, cis-7,10,13,16-docosatetraenoic acid and α-linolenic acid. These free FAs accounted for approximately 95% in all pork samples. The compositions of linoleic acid, palmitic acid and stearic acid in pork LT increased by 121.9%, 66.0% and 55.5%, respectively, after three-month frozen storage. We further compared the sum of saturated FAs (SFAs), mono-unsaturated FAs (MUFAs) and poly-unsaturated FAs (PUFAs) from total FAs (Figure 3C) and free FAs (Figure 3D) along with storage time extension. As shown, MUFAs and PUFAs from total FAs significantly decreased (*p* < 0.01) along with frozen storage time, while SFAs slightly decreased with a non-significant difference (*p* > 0.05). However, all SFAs, MUFA and PUFA from free FAs significantly increased (*p* < 0.01). The results indicated that frozen storage can slow down the lipid hydrolysis but cannot absolutely stop it. Since there were four types of PUFA including ω-3, ω-6, ω-7 and ω-9, we compared the ratios of ω-3 to ω-6, ω-7 to ω-6 and ω-9 to ω-6 in both free and total FAs (Figure 3E,F). The ω-7/ω-6 and ω-9/ω-6 in esterified fatty acids significantly decreased with the extension of storage time, while ω-3/ω-6 was increased. It suggested that lipid oxidation which occurred on ω-7 and ω-9 was faster than that on ω-6.

### 3.3. The Effect of Frozen Storage on Fatty Aldehydes in Pork

**Figure 4:**
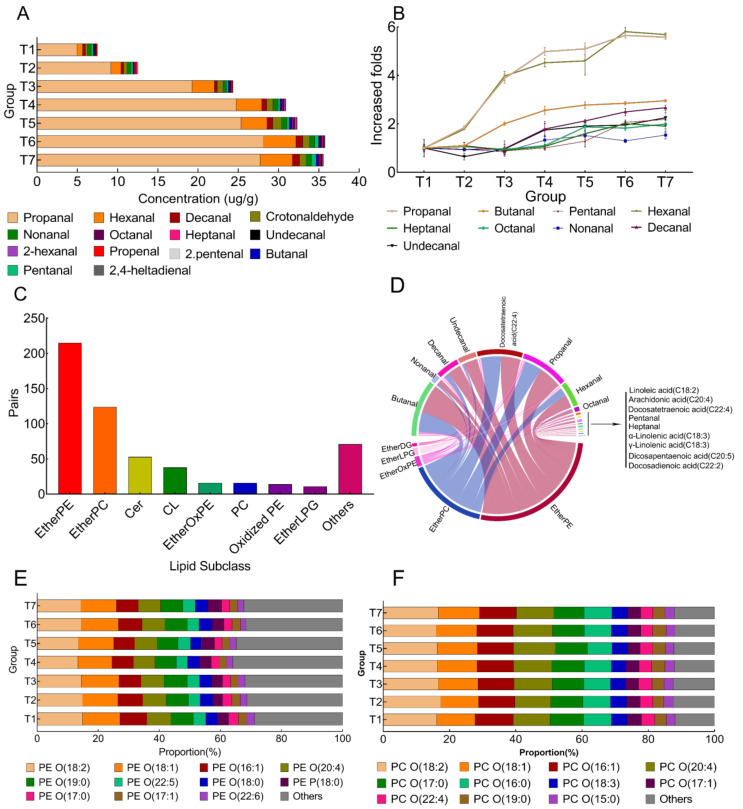
(**A**) The changes in the proportion of fatty aldehydes in pork at different frozen stages; (**B**) Increased fold of saturated fatty aldehydes along with frozen time; Data represented as mean ± std (*n* = 5); (**C**) The highly correlated lipid species with free FAs and fatty aldehydes with R^2^ > 0.9 and *p* < 0.05; (**D**) The chord diagram that correlated ether-linked GPLs with free FAs and fatty aldehydes; (**E**) The major fatty acid compositions in EtherPE of pork lipid; (**F**) The major fatty acids compositions in EtherPC of pork lipid. EtherPE (PE O), ether-linked phosphatidylethanolamine; EtherPC (PC O), ether-linked phosphatidylcholine; Cer, ceramides; CL, cardiolipin; EtherOxPE, ether-linked oxidized phosphatidylethanolamine; PC, phosphatidylcholine; OxPE, oxidized phosphatidylethanolamine; EtherLPC, ether-linked lysophosphatidylcholine; EtherPG, ether-linked phosphatidylglycerol; EtherLPG, ether-linked diacylglycerol; EtherDG, ether-linked diacylglycerol.

PUFAs in lipids were highly susceptible to oxidation in the pork sample exposed to the condition of freezing processes and the corresponding storage [43]. These PUFAs were oxidatively degraded to form secondary oxidative products, aldehydes, which were volatile [44,45]. In this work, we compared the profiles of fatty aldehydes at different stages. The results were summarized in Table 3. Among these quantified aldehydes, propanal and hexanal were the dominant aldehydes followed by decanal and crotonaldehyde in pork LT (Figure 4A). The total amount of aldehydes was significantly increased, and all aldehydes were mainly produced in the first month of freezing. After 3 months of freezing, the total amount of fatty aldehydes increased by around sixfold. Comparing the rate of increase of saturated fatty aldehydes, the increase in propanal and hexanal was faster than that of other fatty aldehydes (Figure 4B). The compositions of unsaturated fatty aldehydes accounted for about 0.5% of total fatty aldehydes.

### 3.4. The Correlation in Changed Tendency of Free FAs and Fatty Aldehydes with Lipid

The correlation coefficient in significantly changed free FAs and aldehydes with lipids (*p* < 0.01) were evaluated using Pearson, shown in Table S7. As shown, a total of 710 highly correlated pairs were found, of which the correlation coefficient (R^2^) was higher than 0.9 and the *p*-value was lower than 0.05. Of these highly correlated pairs, 204 lipids were closely correlated with free FAs and aldehydes (Figure 4C). As shown, these lipids were ascribed to Ether-linked GPLs, especially EtherPE and EtherPC, suggesting that they were the major sources for producing free FAs and aldehydes. Then, the correlation details between Ether-linked GPLs with free FAs and aldehydes were illustrated using the chord diagram (Figure 4D). For free FAs, PUFAs were highly negatively correlated with Ether-linked GPL. Additionally, docosatetraenoic acid (C22:4) was highly negatively correlated with all the Ether-linked GPLs, while other free FAs were negatively correlated with EtherPE and EtherOxPE.

All the aldehydes were mainly negatively correlated with most EtherPE and EtherPC. To further explain the fatty aldehydes variability along with freezing storage, FA compositions in EtherPE and EtherPC were analyzed (Figure 4E,F and Table S8). The EtherPE and EtherPC were mostly composed of MUFAs and PUFAs. Linoleic acid (C18:2), stearic acid (C18:1), cis-9-hexadecenoic acid (C16:1) and arachidonic acid (C20:4) were the main fatty acid structure in EtherPE of pork (Figure 4E). After three-month frozen storage, the concentration of C18:2, C18:1, C16:1 and C20:4 significantly decreased by 45.4%, 45.2%, 53.8% and 46.6%, respectively (Table S8). The decrease in the concentration of C18:2 was negatively correlated with the increase in the content of propanal, butanal, hexanal, decanal, undecanal and crotanaldehyde. A similar trend was also observed for C18:1 and C20:4. In EtherPC (shown in Figure 4F), linoleic acid was the predominant FA structure, followed by stearic acid, cis-9-hexadecenoic acid and arachidonic acid. The contents of C18:2 and C16:1 have negative correlations with those of propanal, butanal, hexanal and crotanaldehyde. These findings suggested that the formation of aldehydes had a strong relationship with UFAs degradation in lipid compositions.

## 4. Discussion

The freezing process is one of the important strategies for maintaining meat quality during long-term storage and transportation. Freezing storage can not only alter physical properties but also produce various biochemical reactions in meat, including lipid oxidation and hydrolysis. In this study, we monitored the variations in lipid species and lipid molecules as well as oxidation products including fatty acids and aldehydes. Non-targeted lipidomic and targeted metabolome were employed. A total of 688 lipids, which was categorized into 28 lipid species, were successfully identified and quantified. Of these lipid species, the top three lipid subclasses were PE (EtherPE and EsterPE), TG and DG, which were consistent with most previous studies [46,47,48]. Notably, the number of quantified EtherPEs and EtherPCs was much higher than that of EsterPEs and EsterPCs. The ether-linked lipids are modified phospholipids containing an alkyl chain attached to the sn1 position by an ether linkage [49]. These lipids are not only important for the organization and stability of lipid raft microdomains, but also serve crucial roles as endogenous antioxidants, such as plasmalogens [50]. Additionally, the oxidative degradation of EtherPE and EtherPC was more serious than in other types of lipids during the frozen storage (Figure 2A,B). This was because UFAs in EtherPE and EtherPC were highly susceptible to oxidization [4,51]. The correlation analysis also indicated that PUFAs were highly negatively correlated with Ether-linked glycerophospholipids. This has been proven by a previous study, which suggested that the antioxidant effect of EtherPE mainly occurred intramolecularly rather than intermolecularly, and it depended on the degree of unsaturation of the sn1 and sn2 side chains [52]. Furthermore, EtherPEs and EtherPCs were highly negatively correlated with most aldehydes such as propanal and hexanal (Figure 4D), which were the oxidative degradation products of UFA in the lipid fraction. Notably, the period of lipid oxidation includes a quick and slow oxidation stage. According to the PCA and OPLS-DA analysis, the decrease in lipids mainly occurred in the first month (T1–T4), and a slight deviation was observed for meat lipids subjected to a longer frozen duration (T4–T7). The increases in free FAs and aldehydes were in the opposite direction of the lipid decrease. Non-enzymatic oxidation of lipids was driven by the production of reactive chemical species contacting with oxygen and light, heme and nonheme iron presence, and gamma-irradiation [44]. These reactive chemical species were boosted after slaughtering through the disruption of cells and redox imbalance due to the absence of redox enzymes under low temperature. In the first month, reactive species were sufficient to promote lipid oxidation. As reactive species were consuming, the lipid oxidation slowed down. This suggested that more visible changes would not be produced and had negative effects on meat quality even when the frozen time was further extended.

To further explore the variations in FA compositions in lipids, both esterified FAs from lipid saponification and free FAs without lipid saponification were quantitatively analyzed. The results indicated that in esterified FAs, the sum of SFAs was higher than that of MUFAs and PUFAs in all pork samples. A slightly progressive decrease in the sum of SFAs along with frozen storage time was observed (Figure 3C). Unlike the sum of SFAs, the sum of esterified MUFAs and PUFAs sharply declined along with frozen storage. It was attributed to both lipolysis and the oxidation of EtherPC and EtherPE. At the same time, free FAs, originated from lipolysis, significantly increased (Figure 3D). The results suggested that the lipolytic enzyme was still able to work with relatively high catalytic efficiency and still underwent the subsequent enzymatic-mediated lipolysis reaction at low temperature (−20 °C). We further analyzed the UFAs oxidation based on the positions of unsaturated bonds with respect to the ratios of ω-3/ω-6, ω-9/ω-6 and ω-7/ω-6 in both free and esterified FAs (Figure 3E,F). The ratio of ω-3/ω-6 slowly increased while the ratios of ω-7/ω-6 and ω-9/ω-6 slowly decreased with the extension of frozen storage time. Many studies showed that ω-3 PUFAs were easily oxidized compared with ω-6 PUFAs since there were more unsaturated bonds in ω-3 PUFAs [53,54,55]. Surprisingly, we found the oxidation occurred at ω-6 PUFAs more than ω-3 PUFAs during the freezing process in this work. The different results may be due to the higher content of ω-6 fatty acids in pig LT (Table 2 and Supplementary Table S6). In addition, lipid oxidation occurring at ω-7 PUFA was more than that at ω-6 fatty acids. Collectively, the above results suggest that frozen storage had a strong influence on the stability of the unsaturated bond of EtherPE and EtherPC, leading to increases in free PUFAs and saturated fatty aldehydes though lipid oxidation.

Generally, the oxidative generation of ω-3 PUFAs could form propanal, acrolein, 2-hexanal and trans, trans-2,4-heptadienal while that of ω-6 PUFA could produce hexanal and heptanal [45]. The oxidative process can affect nutritional content, color, texture, and flavor, and is the most important trigger of meat quality loss. Aldehydes are secondary oxidative products formed by lipid oxidation and strongly influenced by the FA structure in lipids. They can be used to assess the oxidative state of products. For example, Al-Dalali et al. [2] reported that aldehydes including octanal, hexanal, and benzeneacetaldehyde can be used as a potential indicator of meat at different stages of frozen storage. Additionally, aldehydes can be considered a potent class of flavor chemicals that make important contributions to food flavor in both fresh and prepared food products. Sohail et.al reported that aldehydes at a lower concentration were positive to the meat aroma, but high content of aldehydes would provide off-flavor [56]. Propanal is considered as a reliable biomarker in fish meat degradation while hexanal is used as an indicator of flavor degradation in meat [57]. In the current work, 14 aldehydes were successfully quantified, and around 99.5% of aldehydes were saturated aldehydes, which were propanal, butanal and hexanal. Of these saturated aldehydes, propanal and hexanal were the two major aldehydes that rapidly accumulated within the frozen duration (Figure 4B). Notably, total fatty aldehydes increased about sevenfold from the start to end point of the freezing process. To further reveal the correlation between lipid and aldehydes, we also analyzed the FA structure and compositions in EtherPC and EtherPE. The results showed that the lipid oxidation mainly occurred in the UFAs fraction in EtherPE and EtherPE. These UFAs included stearic acid (C18:1), cis-9-hexadecenoic acid (C16:1), linoleic acid (C18:2) and arachidonic acid (C20:4). In livestock and poultry meat, MUFAs are mainly composed of stearic acid while PUFAs consist of linoleic acid and arachidonic acid. Our study showed that octanal and nonanal were the oxidative product of stearic acid in lipid while hexanal and decanal were oxidative products of linoleic acid and arachidonic acid, which was consistent with our results [2,45]. In this work, the formation of volatile aldehydes were degradation products of stearic, cis-9-hexadecenoic, linoleic and arachidonic acid in the lipid fraction.

Noteworthy, we tried to find a new series of compounds that can be used as indicators to evaluate freezing time. Forty-eight prominently changed free FAs and aldehydes were correlated with freezing time, resulting in 23 compounds highly correlated with freezing time with a coefficient of correlation (R^2^) > 0.80 and *p* < 0.05 (Figure 4D and Table S7). There were three compounds for which R^2^ > 0.9, including decanal, cis-11,14-eicosenoic acid and cis-5,8,11,14,17-dicosapentaenoic acid. The robustness and stability of these compounds could be used to calculate the freezing time of the unknown meat sample and are considered to be potential biomarkers to demonstrate multi-batch large samples in our further study.

## 5. Conclusions

A freezing or refrigeration process is a commonly used method to maintain meat quality. In this study, we studied the changing tendency of the lipid subclass, free FAs, total FAs as well as fatty aldehydes along with freezing time. In lipid subclasses, the lipids ether-linked GPL, especially EtherPE and EtherPC, were significantly changed during frozen storage. Additionally, oxidative degradation mainly occurred in EtherPE. As the frozen storage was prolonged, both MUFAs and PUFAs were significantly decreased, suggesting that the lipid oxidation mainly occurred on unsaturated bonds of UFAs. For Free FAs, both SFAs and UFAs, originated from lipolysis, were significantly increased with the frozen time. As the oxidative product of UFAs, fatty aldehydes, especially saturated fatty aldehydes, significantly increased with the extension of freezing time. Based on the above results, the lipid oxidation mainly occurred in the first month of freezing storage. The correlation analysis suggested that decanal, cis-11,14-eicosenoic acid and cis-5,8,11,14,17-dicosapentaenoic acid can be considered as potential biomarkers for freezing storage. Collectively, this study can provide insight into maintaining meat quality during long-time and far-distance food processing.

## Figures and Tables

**Table 1:** The quantified results of different lipid subclasses in pork LT during frozen storage.

Subclass	T1	T2	T3	T4	T5	T6	T7	*p* Value
CAR	7.41 ± 2.3 ^b^	11.82 ± 2.71 ^a^	12.38 ± 1.1 ^a^	10.02 ± 3.42 ^ab^	10.06 ± 2.13 ^ab^	11.66 ± 3.99 ^a^	10.91 ± 2.34 ^ab^	1.18 × 10^−1^
Cer	38.49 ± 5.51 ^a^	35.12 ± 2.95 ^a^	39.94 ± 3.04 ^a^	27.11 ± 5.92 ^b^	25.06 ± 3.01 ^b^	24.83 ± 3.34 ^b^	23.41 ± 5.03 ^b^	3.86 × 10^−7^
CL	33.05 ± 4.42 ^bc^	35.02 ± 3.18 ^b^	44.06 ± 1.87 ^a^	29.33 ± 4.26 ^cd^	25 ± 2.13 ^d^	25.14 ± 6.47 ^d^	19.29 ± 1.93 ^e^	1.43 × 10^−9^
DG	48.37 ± 3.88 ^d^	105.29 ± 15.88 ^ab^	65.86 ± 6.69 ^cd^	125.3 ± 30.05 ^ab^	127.2 ± 37.91 ^a^	128.95 ± 44.27 ^a^	90.61 ± 11.76 ^bc^	6.95 × 10^−5^
EtherDG	5.42 ± 0.71 ^c^	6.63 ± 0.25 ^b^	12.19 ± 0.4 ^a^	5.88 ± 0.38 ^c^	3.66 ± 0.13 ^d^	3.1 ± 0.37 ^e^	3.15 ± 0.18 ^e^	1.08 × 10^−24^
EtherLPC	4.17 ± 0.92 ^bc^	3.01 ± 0.89 ^c^	3.22 ± 0.46 ^c^	3.77 ± 0.91 ^bc^	2.95 ± 0.34 ^c^	6.57 ± 2.01 ^b^	9.81 ± 5.61 ^a^	4.46 × 10^−4^
EtherLPE	6.35 ± 0.92 ^ab^	5.27 ± 1.27 ^ab^	4.54 ± 0.47 ^b^	5 ± 1.18 ^ab^	4.88 ± 1.34 ^ab^	5.84 ± 3.52 ^ab^	7.5 ± 3 ^a^	2.66 × 10^−1^
EtherLPG	2.99 ± 0.33 ^b^	3.4 ± 0.38 ^ab^	2.87 ± 0.34 ^b^	2.73 ± 0.69 ^b^	2.81 ± 0.49 ^b^	4.55 ± 2.29 ^a^	2.52 ± 0.11 ^b^	4.01 × 10^−2^
EtherOxPE	5.37 ± 0.33 ^a^	5.42 ± 0.16 ^a^	5.32 ± 0.25 ^ab^	4.88 ± 1.81 ^abc^	3.42 ± 0.3 ^c^	4.44 ± 1.45 ^abc^	3.84 ± 1.52 ^bc^	2.90 × 10^−2^
EtherPC	106.6 ± 13.38 ^a^	94.35 ± 14.43 ^ab^	84.34 ± 3.68 ^bc^	69.78 ± 10.28 ^cd^	66.05 ± 9.64 ^d^	70.99 ± 17.73 ^cd^	68.31 ± 11.79 ^cd^	4.24 × 10^−5^
EtherPE	114.18 ± 15.74 ^a^	112.56 ± 14.01 ^a^	99.44 ± 7.75 ^a^	80.49 ± 11.14 ^b^	73.46 ± 11 ^b^	74.62 ± 13.74 ^b^	67.22 ± 6.23 ^b^	3.29 × 10^−7^
EtherPG	4.56 ± 0.13 ^bc^	5.34 ± 0.39 ^b^	6.55 ± 0.33 ^a^	4.66 ± 0.47 ^bc^	4.42 ± 0.4 ^c^	4.42 ± 1.28 ^c^	3.11 ± 0.4 ^d^	8.89 × 10^−8^
FA	23.82 ± 3.76 ^ab^	27.33 ± 3.6 ^a^	26.12 ± 2.75 ^ab^	22.26 ± 4.63 ^ab^	21.96 ± 2.68 ^ab^	22.34 ± 6.67 ^ab^	20.08 ± 6.02 ^b^	1.96 × 10^−1^
FAM	26.7 ± 1.7 ^d^	30.66 ± 1.62 ^b^	48.91 ± 3.35 ^a^	27.45 ± 2.15 ^cd^	30.06 ± 0.99 ^bc^	19.26 ± 1.31 ^e^	21.85 ± 3.14 ^e^	2.57 × 10^−17^
LPC	7.47 ± 0.66 ^c^	6.18 ± 1.46 ^bc^	6.54 ± 1.23 ^bc^	6.47 ± 1.89 ^bc^	4.94 ± 0.86 ^c^	7.96 ± 2.34 ^b^	10.61 ± 3.76 ^a^	4.91 × 10^−3^
MG	7.37 ± 0.34 ^c^	7.82 ± 0.27 ^c^	10.89 ± 0.23 ^a^	8.11 ± 0.84 ^c^	9.44 ± 0.6 ^b^	7.76 ± 1.9 ^c^	7.34 ± 0.44 ^c^	1.51 × 10^−6^
Others	23.19 ± 3.33 ^ab^	23.47 ± 3.84 ^ab^	25.42 ± 1.13 ^a^	19.71 ± 3.61 ^abc^	18.93 ± 7.47 ^bc^	19.74 ± 3.85 ^abc^	15.8 ± 2.14 ^c^	1.42 × 10^−2^
OxFA	15.09 ± 2.53 ^a^	16.33 ± 6.48 ^ab^	16.97 ± 0.86 ^ab^	20.64 ± 8.6 ^ab^	12.23 ± 4.65 ^b^	16.42 ± 8.67 ^ab^	9.95 ± 0.6 ^b^	1.16 × 10^−1^
OxPC	5.85 ± 0.63 ^a^	5.38 ± 0.84 ^ab^	4.95 ± 0.21 ^bc^	4.01 ± 0.52 ^d^	3.58 ± 0.47 ^d^	4.22 ± 0.9 ^c^	3.85 ± 0.46 ^d^	9.38 × 10^−6^
OxPE	12.63 ± 2.01 ^a^	13.18 ± 1.56 ^a^	12.92 ± 1.24 ^a^	9.51 ± 1.55 ^c^	9.59 ± 1.3 ^bc^	11.47 ± 1.85 ^ab^	9.04 ± 1.14 ^bc^	1.95 × 10^−4^
OxTG	4.29 ± 0.85	3.86 ± 0.24	4.51 ± 1.31	3.93 ± 1.76	3.45 ± 0.59	4.32 ± 2.27	3.57 ± 0.27	8.02 × 10^−1^
PC	43.96 ± 4.47 ^a^	35.01 ± 4.35 ^b^	34.54 ± 2.09 ^b^	32.41 ± 5.08 ^b^	30.96 ± 4.82 ^b^	30.13 ± 5.6 ^b^	32.53 ± 4.55 ^b^	1.04 × 10^−3^
PE	10.15 ± 0.54 ^a^	8.91 ± 1.05 ^ab^	8.62 ± 0.94 ^b^	8.03 ± 1.13 ^b^	8.46 ± 1.45 ^b^	8.11 ± 1.04 ^b^	7.9 ± 0.62 ^b^	2.39 × 10^−2^
PG	3.74 ± 0.33 ^b^	4.29 ± 0.71 ^b^	5.51 ± 0.2 ^a^	3.73 ± 0.71 ^b^	3.98 ± 1.09 ^b^	3.51 ± 0.63 ^b^	2.64 ± 0.38 ^c^	1.36 × 10^−5^
PS	3.05 ± 0.79	2.41 ± 0.94	3.3 ± 0.47	2.53 ± 0.76	3.74 ± 4.18	3.68 ± 1.46	1.96 ± 0.55	6.37 × 10^−1^
SM	18.59 ± 3.76 ^a^	16.66 ± 5.5 ^ab^	12.99 ± 1.2 ^bc^	12.56 ± 2.53 ^bc^	9.12 ± 2.84 ^c^	15.7 ± 4.57 ^ab^	15.6 ± 3.9 ^ab^	9.35 × 10^−3^
TG	34.85 ± 7.32 ^a^	21.85 ± 3.85 ^b^	40.4 ± 14.65 ^ab^	44.74 ± 20.45 ^ab^	38.37 ± 10.8 ^a^	42.31 ± 28.63 ^ab^	20.39 ± 3.24 ^b^	9.42 × 10^−2^
VAE	8.67 ± 0.5 ^abcd^	7.87 ± 1.46 ^cd^	8.22 ± 0.12 ^bc^	9.47 ± 1.16 ^abc^	9.84 ± 1.82 ^ab^	7.24 ± 0.74 ^d^	10.1 ± 1.91 ^a^	9.68 × 10^−3^

Data represented as mean ± standard deviation (std) (*n* = 5). Data in the same row for one parameter with different lowercase letters (a–e) show a significance difference of (*p* < 0.05). CAR, acylcarnitine; Cer, ceramides; CL, cardiolipin; DG, diacylglycerol; EtherDG, ether-linked diacylglycerol; EtherLPC, ether-linked lysophosphatidylcholine; EtherLPE, ether-linked lysophosphatidylethanolamine; EtherLPG, ether-linked lysophosphatidylglycerol; EtherOxPE, ether-linked oxidized phosphatidylethanolamine; EtherPC, ether-linked phosphatidylcholine; EtherPE, ether-linked phosphatidylethanolamine; EtherPG, ether-linked phosphatidylglycerol; FA, free fatty acid; FAM, fatty amides; LPC, lysophophatidylcholine ; MG, monoacylglycerol; OxFA, oxidized fatty acid; OxPC, oxidized phosphatidylcholine; OxPE, oxidized phosphatidylethanolamine; OxTG, oxidized triacylglycerols; PC, phosphatidylcholine; PE, phosphatidylethanolamine; PG, phosphatidylglycerol; PS, phosphatidylserine; SM, sphingomyelin; TG, triacylglycerols; VAE, vitamin A fatty acid ester.

**Table 2:** The quantified results of free fatty acid of pork at different frozen storage stages (mg/g).

Compounds	T1	T2	T3	T4	T5	T6	T7	*p* Value
Hexanoic acid (C6:0)	41.95 ± 12.10 ^b^	45.95 ± 8.53 ^b^	45.01 ± 9.66 ^b^	36.98 ± 10.53 ^b^	50.87 ± 15.91 ^b^	52.79 ± 18.52 ^b^	92.25 ± 22.24 ^a^	5.20 × 10^−5^
Heptanoic acid (C7:0)	8.49 ± 0.78 ^b^	6.47 ± 0.83 ^b^	8.78 ± 2.01 ^b^	9.61 ± 2.28 ^b^	9.46 ± 3.16 ^b^	8.41 ± 3.28 ^b^	16.00 ± 2.45 ^a^	2.77 × 10^−5^
Octanoic acid (C8:0)	12.14 ± 3.90 ^b^	12.94 ± 1.35 ^b^	19.66 ± 5.56 ^b^	16.97 ± 4.77 ^b^	18.72 ± 7.34 ^b^	20.82 ± 7.64 ^b^	35.23 ± 5.86 ^a^	1.04 × 10^−5^
Nonanoic acid (C9:0)	67.85 ± 8.54 ^b^	66.69 ± 8.96 ^b^	89.75 ± 12.48 ^ab^	90.74 ± 21.71 ^ab^	72.24 ± 14.49 ^b^	66.89 ± 22.09 ^b^	125.5 ± 40.09 ^a^	9.76 × 10^−4^
Decanoic acid (C10:0)	7.18 ± 1.19 ^b^	7.91 ± 2.77 ^b^	8.08 ± 1.21 ^b^	10.42 ± 2.40 ^ab^	7.37 ± 1.63 ^b^	8.40 ± 1.31 ^ab^	12.31 ± 2.47 ^a^	2.35 × 10^−3^
Undecanoic acid (C11:0)	2.24 ± 0.23 ^b^	2.47 ± 0.70 ^b^	2.05 ± 0.14 ^b^	2.31 ± 0.97 ^b^	2.06 ± 0.42 ^b^	3.02 ± 1.56 ^ab^	4.17 ± 0.86 ^a^	3.98 × 10^−3^
Dodecanoic acid (C12:0)	40.8 ± 3.21 ^c^	42.88 ± 5.67 ^c^	43.67 ± 8.91 ^c^	66.35 ± 5.15 ^ac^	46.98 ± 8.69 ^bc^	55.1 ± 12.27 ^abc^	60.44 ± 8.66 ^ab^	7.05 × 10^−5^
Tridecanoic acid (C13:0)	9.91 ± 1.44 ^a^	3.95 ± 0.32 ^c^	4.88 ± 0.69 ^bc^	3.80 ± 0.61 ^c^	2.75 ± 0.75 ^c^	4.60 ± 1.05 ^bc^	6.61 ± 1.83 ^b^	5.40 × 10^−10^
Myristic acid (C14:0)	215.64 ± 14.77 ^b^	190.81 ± 76.93 ^b^	190.73 ± 28.7 ^b^	251.99 ± 31.85 ^ab^	190.77 ± 31.12 ^b^	267.99 ± 49.63 ^ab^	328.75 ± 69.32 ^a^	4.46 × 10^−4^
Pentadecanoic acid (C15:0)	59.82 ± 7.48 ^a^	28.80 ± 3.94 ^c^	46.21 ± 6.7 ^b^	53.51 ± 6.58 ^ab^	41.49 ± 3.93 ^b^	46.32 ± 7.16 ^b^	61.44 ± 7.16 ^a^	6.19 × 10^−8^
Palmitic acid (C16:0)	2285.23 ± 151.17 ^cd^	2145.11 ± 427.41 ^d^	2288.36 ± 210.11 ^cd^	2702.32 ± 276.34 ^bc^	2276.38 ± 72.14 ^cd^	2904.48 ± 227.19 ^b^	3792.98 ± 283.38 ^a^	3.86 × 10^−10^
cis-9-Hexadecenoic acid (C16:1)	190.44 ± 12.6 ^cd^	178.76 ± 35.62 ^d^	190.7 ± 17.51 ^cd^	225.19 ± 23.03 ^bc^	189.7 ± 6.01 ^cd^	242.04 ± 18.93 ^b^	316.08 ± 23.61 ^a^	3.86 × 10^−10^
Heptadecanoic acid (C17:0)	25.74 ± 3.07 ^cd^	24.69 ± 1.47 ^d^	36.20 ± 3.18 ^bc^	38.16 ± 6.11 ^b^	36.26 ± 4.96 ^bc^	42.02 ± 8.06 ^b^	80.19 ± 8.56 ^a^	3.38 × 10^−14^
Stearic acid (C18:0)	1493.30 ± 134.63 ^c^	1404.80 ± 169.27 ^c^	1624.05 ± 35.98 ^bc^	1863.03 ± 217.15 ^b^	1509.55 ± 110.36 ^c^	1862.05 ± 151.18 ^b^	2321.65 ± 161.52 ^a^	1.54 × 10^−9^
Elaidic acid (C18:1)	64.31 ± 4.25 ^cd^	51.33 ± 10.78 ^d^	61.39 ± 11.00 ^cd^	77.7 ± 10.49 ^b^	63.13 ± 11.16 ^cd^	86.84 ± 14.30 ^b^	125.94 ± 10.44 ^a^	1.92 × 10^−10^
cis-9-Octadecenoic acid (C18:1)	990.02 ± 111.94 ^d^	1283.14 ± 216.31 ^cd^	1412.77 ± 180.39 ^bcd^	1623.19 ± 370.00 ^bc^	1654.44 ± 202.12 ^bc^	1837.89 ± 171.57 ^b^	2939.24 ± 414.91 ^a^	9.89 × 10^−11^
Linoleic acid (C18:2)	4651.50 ± 1994.05 ^cd^	4525.17 ± 516.76 ^d^	4937.29 ± 1073.83 ^cd^	5858.23 ± 1474.05 ^cd^	7379.44 ± 1988.49 ^bc^	8856.42 ± 951.04 ^ab^	10,323.84 ± 1177.47 ^a^	5.18 × 10^−7^
α-Linolenic acid (C18:3)	93.44 ± 31.32 ^c^	91.02 ± 14.83 ^c^	93.66 ± 16.33 ^c^	112.52 ± 20.26 ^bc^	128.92 ± 25.74 ^bc^	155.86 ± 2.97 ^ab^	191.37 ± 28.10 ^a^	1.21 × 10^−7^
γ-Linolenic acid (C18:3)	32.11 ± 14.81 ^c^	34.39 ± 6.47 ^c^	37.26 ± 5.30 ^c^	42.71 ± 11.99 ^c^	51.08 ± 11.39 ^bc^	70.27 ± 12.73 ^ab^	84.4 ± 15.18 ^a^	1.86 × 10^−7^
Nonadecanoic acid (C19:0)	17.94 ± 3.52 ^b^	14.87 ± 1.25 ^b^	21.68 ± 2.71 ^b^	21.68 ± 0.90 ^b^	22.73 ± 4.38 ^b^	21.73 ± 3.62 ^b^	43.12 ± 8.27 ^a^	8.89 × 10^−10^
Arachidic acid (C20:0)	13.21 ± 1.79 ^c^	10.38 ± 3.18 ^c^	14.28 ± 0.66 ^b^	19.86 ± 3.28 ^ab^	13.32 ± 4.12 ^c^	13.8 ± 2.15 ^c^	23.48 ± 3.34 ^a^	7.65 × 10^−7^
cis-11-Eicosenoic acid (C20:1)	49.34 ± 9.78 ^b^	59.72 ± 24.38 ^b^	58.7 ± 10.20 ^b^	69.85 ± 21.63 ^b^	70.46 ± 14.56 ^b^	80.04 ± 12.7 ^b^	120.94 ± 11.93 ^a^	3.09 × 10^−6^
cis-11,14-Eicosenoic acid (C20:2)	31.03 ± 2.94 ^d^	41.47 ± 5.01 ^cd^	46.85 ± 5.68 ^c^	54.14 ± 9.29 ^bc^	56.34 ± 6.47 ^bc^	64.19 ± 4.83 ^b^	94.20 ± 13.21 ^a^	5.73 × 10^−12^
cis-8,11,14-Eicosenoic acid (C20:3)	13.99 ± 4.18 ^b^	12.41 ± 2.72 ^b^	12.46 ± 2.96 ^b^	13.09 ± 1.43 ^b^	13.41 ± 1.14 ^b^	12.22 ± 2.68 ^b^	28.95 ± 5.19 ^a^	1.67 × 10^−8^
cis-11,14,17-Eicosenoic acid (C20:3)	180.19 ± 39.77 ^bcd^	148.48 ± 14.84 ^cd^	137.84 ± 6.01 ^d^	190.01 ± 43.23 ^bcd^	235.35 ± 70.14 ^bc^	265.19 ± 35.19 ^b^	378.32 ± 74.27 ^a^	7.58 × 10^−8^
Arachidonic acid (C20:4)	1432.37 ± 339.35 ^d^	1511.11 ± 154.33 ^d^	1515.61 ± 175.7 ^d^	1952.3 ± 383.42 ^cd^	2590.8 ± 522.5 ^bc^	3007.3 ± 235.77 ^ab^	3723.1 ± 820.65 ^a^	3.98 × 10^−9^
cis-5,8,11,14,17-Dicosapentaenoic acid (C20:5)	59.82 ± 13.49 ^d^	70.2 ± 8.95 ^d^	71.98 ± 16.64 ^d^	93.55 ± 27.53 ^cd^	135.13 ± 32.90 ^bc^	173.17 ± 30.49 ^ab^	217.17 ± 27.59 ^a^	4.16 × 10^−11^
Heneicosanoic acid (C21:0)	2.62 ± 1.41 ^bc^	0.68 ± 0.09 ^c^	1.17 ± 0.11 ^bc^	3.16 ± 2.47 ^bc^	3.88 ± 1.90 ^b^	1.06 ± 0.36 ^c^	7.66 ± 1.38 ^a^	1.42 × 10^−7^
Behenic acid (C22:0)	3.08 ± 1.14 ^c^	2.01 ± 0.47 ^c^	3.43 ± 0.35 ^bc^	3.63 ± 0.59 ^b^	3.26 ± 0.54 ^bc^	3.68 ± 0.60 ^b^	8.38 ± 1.16 ^a^	5.28 × 10^−12^
cis-13-Docosenoic acid (C22:1)	14.05 ± 3.84 ^b^	7.99 ± 2.67 ^b^	10.13 ± 2.62 ^b^	11.1 ± 4.91 ^b^	9.69 ± 3.75 ^b^	11.79 ± 1.98 ^b^	31.16 ± 4.34 ^a^	6.29 × 10^−10^
cis-13,16-Docosadienoic acid (C22:2)	3.26 ± 0.87 ^b^	5.95 ± 2.21 ^b^	4.16 ± 1.20 ^b^	3.75 ± 1.37 ^b^	5.72 ± 1.53 ^b^	5.33 ± 1.78 ^b^	12.72 ± 1.80 ^a^	5.23 × 10^−9^
cis-13,16,19-Docosatrienoic acid (C22:3)	6.15 ± 1.57 ^d^	11.03 ± 1.48 ^c^	11.12 ± 1.26 ^c^	8.69 ± 3.54 ^cd^	22.45 ± 2.75 ^a^	16.88 ± 1.50 ^b^	23.51 ± 2.63 ^a^	3.65 × 10^−13^
cis-7,10,13,16-Docosatetraenoic acid (C22:4)	120.77 ± 31.41 ^e^	175.54 ± 9.91 ^cde^	163.89 ± 10.47 ^de^	224.38 ± 46.00 ^bcd^	259.61 ± 39.24 ^abc^	332.34 ± 42.64 ^a^	279.61 ± 78.75 ^ab^	1.48 × 10^−7^
cis-7,10,13,16,19-Docosatetraenoic acid (C22:5)	23.36 ± 10.40 ^c^	22.46 ± 3.62 ^c^	24.21 ± 4.66 ^c^	32.45 ± 9.60 ^bc^	42.84 ± 15.37 ^bc^	50.74 ± 7.99 ^ab^	71.71 ± 17.40 ^a^	2.79 × 10^−7^
cis-4,7,10,13,16,19-Docosatetraenoic acid (C22:6)	90.5 ± 37.1 ^c^	75.54 ± 8.10 ^c^	88.9 ± 27.58 ^c^	111.24 ± 35.63 ^bc^	153.63 ± 17.95 ^ab^	175.62 ± 68.84 ^ab^	210.97 ± 56.63 ^a^	5.22 × 10^−5^
Lignoceric acid (C24:0)	15.49 ± 4.85 ^b^	7.49 ± 1.15 ^c^	12.31 ± 1.41 ^bc^	14.4 ± 3.19 ^b^	13.78 ± 3.78 ^bc^	16.35 ± 3.99 ^b^	28.09 ± 3.45 ^a^	2.55 × 10^−8^
cis-15-Tetracosenoic acid (C24:1)	7.13 ± 1.20 ^c^	8.15 ± 0.80 ^c^	9.71 ± 1.57 ^bc^	9.34 ± 2.00 ^bc^	12.21 ± 2.58 ^b^	12.7 ± 2.01 ^b^	25.21 ± 1.71 ^a^	1.21 × 10^−14^
Hexacosanoic acid (C26:0)	14.3 ± 3.04 ^b^	7.11 ± 1.55 ^b^	10.05 ± 1.56 ^b^	12.19 ± 3.03 ^b^	9.92 ± 2.55 ^b^	13.28 ± 5.57 ^b^	28.91 ± 6.96 ^a^	5.52 × 10^−8^
Octacosanoic acid (C28:0)	3.69 ± 1.24 b	1.68 ± 0.25 b	3.11 ± 0.52 b	2.59 ± 0.81 b	2.94 ± 2.20 b	3.74 ± 0.98 b	16.83 ± 4.61 a	1.79 × 10^−11^
Melissic acid (C30:0)	6.22 ± 3.50 ^b^	1.41 ± 0.19 ^b^	3.64 ± 1.50 ^b^	1.62 ± 0.42 ^b^	2.78 ± 2.71 ^b^	1.55 ± 1.07 ^b^	22.54 ± 4.63 ^a^	3.58 × 10^−13^
SFA	4346.85 ± 296.53 ^d^	4029.1 ± 605.20 ^d^	4477.09 ± 294.29 ^cd^	5225.32 ± 378.14 ^bc^	4337.51 ± 187.17 ^d^	5418.07 ± 476.1 ^b^	7116.53 ± 554.60 ^a^	3.18 × 10^−11^
PUFA	6738.5 ± 2465.51 ^d^	6724.76 ± 609.61 ^d^	7145.23 ± 1262.16 ^d^	8697.06 ± 1909.39 ^cd^	11,074.72 ± 2646.61 ^bc^	13,185.52 ± 1300.82 ^ab^	15,639.87 ± 1737.61 ^a^	1.43 × 10^−8^
MUFA	3410.08 ± 193.36 ^d^	3555.44 ± 640.99 ^d^	3841.04 ± 338.74 ^cd^	4493.5 ± 217.81 ^bc^	4086.3 ± 230.95 ^bcd^	4933.73 ± 393.69 ^b^	7035.46 ± 688.71 ^a^	1.00 × 10^−12^
UFA	10,148.57 ± 2540.65 ^d^	10,280.20 ± 1057.40 ^d^	10,986.27 ± 1300.68 ^d^	13,190.56 ± 1969.59 ^cd^	15,161.02 ± 2644.33 ^bc^	18,119.25 ± 1052.51 ^b^	22,675.33 ± 1517.95 ^a^	2.30 × 10^−11^
Total FA	14,495.42 ± 2602.75 ^d^	14,309.30 ± 1652.90 ^d^	15,463.37 ± 1512.93 ^d^	18,415.88 ± 2090.33 ^cd^	19,498.54 ± 2778.78 ^c^	23,537.32 ± 923.82 ^b^	29,791.87 ± 1297.66 ^a^	9.07 × 10^−13^
ω3/ω6	0.08 ± 0.02	0.07 ± 0.00	0.06 ± 0.00	0.07 ± 0.00	0.07 ± 0.01	0.07 ± 0.00	0.08 ± 0.00	1.95 × 10^−1^
ω7/ω6	0.42 ± 0.02	0.34 ± 0.06	0.35 ± 0.05	0.34 ± 0.07	0.23 ± 0.05	0.24 ± 0.04	0.26 ± 0.05	2.23 × 10^−2^
ω9/ω6	0.21 ± 0.12	0.22 ± 0.04	0.24 ± 0.07	0.23 ± 0.08	0.19 ± 0.07	0.17 ± 0.03	0.23 ± 0.05	6.87 × 10^−1^

SFA means total saturated fatty acids. PUFA means total poly-unsaturated fatty acids. MUFA means total mono-unsaturated fatty acids. UFA means total unsaturated fatty acids. ω3/ω6, ω7/ω6 and ω9/ω6 mean the ratios of ω3 to ω6 fatty acids, ω7 to ω6 fatty acids and ω9 to ω6 fatty acids. Data represented as mean ± std (*n* = 5). Data in the same row for one parameter with a different lowercase letter (a–e) have a significant difference of (*p* < 0.05).

**Table 3:** The changes in fatty aldehyde compositions in pork during frozen storage (ng/g).

Compounds	T1	T2	T3	T4	T5	T6	T7	*p* Value
Propanal	49.69 ± 1.22 ^c^	91.8 ± 3.30 ^c^	192.49 ± 9.21 ^c^	247.52 ± 7.98 ^b^	253.28 ± 1.23 ^b^	280.91 ± 5.56 ^a^	277.19 ± 4.13 ^a^	2.85 × 10^−30^
Butanal	0.59 ± 0.01 ^d^	0.64 ± 0.02 ^d^	1.18 ± 0.04 ^c^	1.50 ± 0.10 ^b^	1.63 ± 0.01 ^a^	1.68 ± 0.04 ^a^	1.74 ± 0.03 ^a^	2.74 × 10^−24^
Pentanal	2.37 ± 0.24 ^bc^	2.62 ± 0.27 ^bc^	2.09 ± 0.41 ^c^	2.38 ± 0.16 ^bc^	3.04 ± 0.05 ^b^	4.89 ± 0.21 ^a^	5.18 ± 0.67 ^a^	1.73 × 10^−13^
Hexanal	7.01 ± 0.26 ^e^	12.49 ± 0.15 ^d^	27.86 ± 1.29 ^c^	31.72 ± 1.17 ^b^	32.27 ± 0.41 ^b^	40.73 ± 1.20 ^a^	39.89 ± 0.49 ^a^	1.57 × 10^−23^
Heptanal	0.78 ± 0.06 ^c^	0.86 ± 0.11 ^c^	0.71 ± 0.08 ^c^	0.83 ± 0.06 ^c^	1.25 ± 0.01 ^b^	1.57 ± 0.07 ^a^	1.49 ± 0.20 ^ab^	8.92 × 10^−13^
Octanal	1.28 ± 0.05 ^b^	1.36 ± 0.05 ^b^	1.22 ± 0.1 ^b^	1.41 ± 0.24 ^b^	2.4 ± 0.02 ^a^	2.34 ± 0.14 ^a^	2.55 ± 0.25 ^a^	1.15 × 10^−14^
Nonanal	4.73 ± 0.75 ^b^	4.48 ± 0.32 ^b^	4.02 ± 0.49 ^b^	6.27 ± 0.55 ^a^	7.19 ± 0.07 ^a^	6.13 ± 0.33 ^a^	7.28 ± 0.76 ^a^	9.60 × 10^−9^
Decanal	3.37 ± 0.43 ^d^	3.17 ± 0.10 ^d^	3.29 ± 1.09 ^d^	6.02 ± 1.06 ^c^	7.16 ± 0.02 ^bc^	8.41 ± 0.52 ^ab^	9.00 ± 0.34 ^a^	4.80 × 10^−16^
Undecanal	0.59 ± 0.21 ^a^	0.38 ± 0.08 ^a^	0.57 ± 0.12 ^a^	1.04 ± 0.16 ^b^	1.13 ± 0.01 ^b^	1.16 ± 0.09 ^b^	1.34 ± 0.19 ^b^	1.55 × 10^−11^
Propenal	1.35 ± 0.29 ^b^	1.88 ± 0.16 ^a^	1.16 ± 0.05 ^b^	0.95 ± 0.11 ^bc^	0.67 ± 0.04 ^cd^	0.45 ± 0.08 ^d^	0.7 ± 0.05 ^cd^	1.34 × 10^−10^
Crotonaldehyde	2.59 ± 0.08 ^e^	4.56 ± 0.22 ^d^	7.65 ± 0.31 ^c^	7.86 ± 0.47 ^c^	10.41 ± 0.02 ^a^	8.66 ± 0.18 ^b^	8.13 ± 0.20 ^bc^	8.71 × 10^−27^
2-Pentenal	0.04 ± 0.01	0.06 ± 0.01	0.04 ± 0.00	0.06 ± 0.00	0.08 ± 0.00	0.07 ± 0.00	0.07 ± 0.00	8.30 × 10^−14^
2-Hexanal	0.5 ± 0.03 ^e^	0.66 ± 0.02 ^e^	1.07 ± 0.05 ^d^	1.53 ± 0.12 ^b^	2.55 ± 0.02 ^a^	0.64 ± 0.01 ^e^	1.28 ± 0.05 ^c^	3.28 × 10^−23^
2,4-Heltadienal	0.03 ± 0.01 ^c^	0.16 ± 0.00 ^b^	0.10 ± 0.01 ^b^	0.05 ± 0.00 ^b^	0.12 ± 0.00 ^b^	0.03 ± 0.00 ^c^	0.06 ± 0.00 ^b^	2.63 × 10^−14^

Data represented as mean ± std (*n* = 5). Data in the same row for one parameter with different lowercase letter (a–e) with a significant difference (*p* < 0.05).

## Data Availability

The data are contained within article and Supplementary Materials.

## Supplementary Materials

The following supporting information can be downloaded at: https://www.mdpi.com/article/10.3390/metabo12100977/s1. Table S1. Lipids identified in positive mode; Table S2. Lipids identified in negative mode; Table S3. Lipids and lipid species identified in pig LT during freezing process; Table S4. Significant changed lipids in pork during freezing process (*p* < 0.05); Table S5 Significant changed lipid classes in frozen meat during freezing process (*p* < 0.05); Table S6 Effect of frozen storage duration on total fatty acids compositions (μg/g) of pork; Table S7. The correlation analysis among lipids, fatty aldehydes and free fatty acids; Table S8. Effect of frozen storage on fatty acids compositions in EtherPE and EtherPC.
